# Bacteriological Profiles of Post-mortem Blood Cultures and Their Relationship to the Cause of Death: A Study of 100 Autopsies

**DOI:** 10.7759/cureus.104988

**Published:** 2026-03-10

**Authors:** Kunal Gaba, Kuldeep Kumar, Rahul Kaushik, Vidhi Sachdeva, Kamal Singla, Treasa James, Pushkar Vasisht, Madhu Sharma

**Affiliations:** 1 Forensic Medicine and Toxicology, Shree Guru Gobind Singh Tricentenary (SGT) Hospital and Research Institute, Gurugram, IND; 2 Forensic Medicine and Toxicology, Al-Falah School of Medical Sciences and Research Centre, Faridabad, IND; 3 Forensic Medicine, Pandit Bhagwat Dayal Sharma Post Graduate Institute of Medical Sciences, Rohtak, IND; 4 Forensic Medicine and Toxicology, All India Institute of Medical Sciences (AIIMS) Bathinda, Bathinda, IND; 5 Pharmacology, Employees' State Insurance Corporation (ESI) Medical College and Hospital, Faridabad, IND; 6 Forensic Medicine, All India Institute of Medical Sciences (AIIMS) Bathinda, Bathinda, IND; 7 Microbiology, SKS Hospital Medical College and Research Centre, Mathura, IND; 8 Microbiology, Pandit Bhagwat Dayal Sharma Post Graduate Institute of Medical Sciences, Rohtak, IND

**Keywords:** blood culture profile, cause of death, forensic microbiology, medico-legal autopsy, postmortem

## Abstract

Background: The use of post-mortem blood culture as a diagnostic tool in medico-legal autopsies is limited. Differentiating true infection from contamination or post-mortem bacterial translocation remains a challenge. This study aimed to evaluate the bacteriological profiles of post-mortem blood cultures and their association with the cause of death.

Methods: A cross-sectional observational study was conducted on 100 autopsy cases with unnatural causes of death and a post-mortem interval of less than 24 hours. Cases of septicaemia and putrefied bodies were excluded. Heart blood samples were collected aseptically and cultured using standard microbiological techniques. Associations between culture results and cause of death, time since death, and hospital stay were analysed using the chi-square test.

Results: Of the 100 cases, 66% showed bacterial growth, while 34% were sterile. Single-organism growth was observed in 92.4% of positive cultures. The most commonly isolated organism was *Klebsiella pneumoniae* (42.4%), followed by *Enterobacter* spp. (13.6%) and *Acinetobacter baumannii* (13.1%). Blood cultures were positive in nearly all cases of death due to disease and in more than two-thirds of polytrauma cases. A statistically significant association was found between the cause of death and culture positivity (p = 0.043). No significant association was observed between the time from death to autopsy (p = 0.642) or the duration of hospital stay (p = 0.988).

Conclusion: The cause of death significantly influences post-mortem blood culture results, with higher positivity in disease-related and polytrauma deaths. Time since death and prior hospitalisation did not affect culture outcomes. Careful sampling within 24 hours can yield meaningful bacteriological findings that may aid in determining the cause of death.

## Introduction

In cases of medico-legal autopsy, part or all of the internal organs and viscera are sometimes preserved. Apart from these, blood is sometimes collected and sent for post-mortem microbiological testing, an essential but seldom-used diagnostic tool. Post-mortem blood culture is commonly used to detect infectious bacteria and correlate with the cause of death [1]. According to a review published on post-mortem bacteriology by Morris et al., there are, in theory, four mechanisms through which bacteria can appear in post-mortem cultures: (1) invasion during life (genuine positive); (2) agonal spread, i.e., bacterial invasion during the dying process or during artificial maintenance of circulation and respiration at resuscitation; (3) post-mortem translocation due to migration from the mucosal surface into the blood and body tissues; and (4) contamination, where bacteria are introduced into the blood, cerebrospinal fluid (CSF), or tissues during sampling. It is essential to obtain the sample within 24 hours of death, as normal intestinal and cutaneous flora may contaminate the blood by migrating from the mucosal surfaces. Sterile sampling is essential to rule out contamination [2]. However, the rate of contamination and the extent of post-mortem spread of organisms are the two most important factors influencing variations in post-mortem blood culture results. If the majority of isolates are due to contamination or post-mortem spread, then the cultures are a waste of time and resources. For this reason, heart blood and CSF fluid are considered to be the most valuable substances for post-mortem bacteriological cultures [3].

In addition to contamination, one should also consider the normal microbial flora of the human body. A diverse microbial flora is associated with the skin and mucous membranes of every human being from shortly after birth until death. The human body, which contains about 10 cells, routinely harbours about 10^14 ^bacteria. This bacterial population constitutes the normal microbial flora. They may harm the host (by causing dental caries, abscesses, or other infectious diseases) or may exist as commensals (inhabiting the host for long periods without causing detectable harm or benefit) [4]. Thus, in post-mortem microbiology, it is essential to differentiate bacteria contributing to the cause of death from the body's normal flora and contaminants, as these may alter autopsy findings and ultimately affect the cause of death.

There are very few studies on this topic worldwide. As previously mentioned, using splenic tissue as a culture sample carries a risk of contamination. Therefore, in this study, only heart blood samples were collected following proper sterile sample collection procedures. The wide variability in post-mortem blood culture positivity reported across studies has been attributed to multiple mechanisms, including true antemortem bacteremia, agonal spread, post-mortem bacterial translocation, and contamination during sampling or processing. Recent advances in forensic microbiology and microbial forensics, including quantitative and molecular approaches, have further highlighted the complexity of interpreting post-mortem cultures and the need for cautious, context-based evaluation of microbiological findings in medico-legal autopsies [2-17]. Therefore, the present study was undertaken to evaluate the bacteriological profile and pattern of bacterial growth in post-mortem blood cultures and to assess their correlation with the cause of death in autopsy cases.

## Materials and methods

Study design and setting

The present study was a cross-sectional observational study conducted in the Department of Forensic Medicine in collaboration with the Department of Microbiology at Pandit Bhagwat Dayal Sharma Post Graduate Institute of Medical Sciences (PGIMS), Rohtak, Haryana, India, a tertiary care teaching hospital. A total of 100 autopsy cases were included in the study. The cases consisted of deceased individuals with an unnatural cause of death, where the time since death (TSD) was within 24 hours. Cases were excluded if the cause of death was septicaemia, if the TSD exceeded 24 hours, or if the bodies showed signs of putrefaction. All legal and ethical formalities required for medico-legal autopsies were completed before sample collection. The study obtained approval from the Institutional Ethics Committee of Pandit Bhagwat Dayal Sharma Post Graduate Institute of Medical Sciences, Rohtak, prior to commencement (approval no.: IEC/Th/18/Forensic04).

Sample collection procedure

Blood culture samples were collected immediately after opening the chest cavity during the autopsy. In each case, a single heart blood sample was collected aseptically from one cardiac chamber or a major intrathoracic vessel to maintain uniformity and minimise contamination. Before sample collection, the heart and major vessels were decontaminated with cotton wool soaked in 70% alcohol. The proposed puncture site was further disinfected with a swab stick containing 10% povidone iodine. A 20 ml sterile syringe fitted with an 18-20 gauge needle was inserted into the target cardiac ventricle or major vessel, and approximately 5 ml of blood was aspirated. The collected blood was immediately injected into sterile culture vials containing 50 ml of glucose broth, resulting in a blood-to-broth ratio of approximately 1:5. This ratio was maintained to dilute inhibitory substances and ensure adequate nutrient availability for bacterial growth. The inoculated culture bottles were transported to the Department of Microbiology as soon as possible for further processing.

Microbiological processing

All cultures were incubated under aerobic conditions at 37°C, as anaerobic culture facilities were not employed in the present study. Subcultures were performed after 24 hours of incubation and again on the seventh day using sheep blood agar and MacConkey agar media. Subculturing on the seventh day was performed to facilitate the detection of slow-growing organisms or low bacterial loads that may be encountered in post-mortem samples. Cultures were considered sterile if no bacterial growth was observed after seven days of incubation. Bacterial colonies grown on blood agar and MacConkey's agar were further analysed for identification. Organisms were identified based on colony morphology, staining characteristics, and biochemical reactions, following standard microbiological procedures. All microbiological procedures were carried out following standard laboratory protocols, with routine internal quality control practices as per institutional microbiology laboratory guidelines.

The study did not involve the use of specialised medical devices or novel technologies. Blood sample collection and microbiological processing were performed using routine, widely accepted clinical and laboratory methods. Therefore, manufacturer details were not applicable.

Data analysis

All collected data were entered into Microsoft Excel (Microsoft Corporation, Redmond, Washington) and analysed using IBM SPSS Statistics for Windows, Version 21 (Released 2012; IBM Corp., Armonk, New York). Descriptive statistics were used to summarise the data. Categorical variables such as age group, sex, cause of death, duration of hospital stay, and blood culture results were expressed as frequencies and percentages. The association between bacterial growth and various demographic and clinical variables was assessed using the chi-square test. A p-value of less than 0.05 was considered statistically significant. Results were presented in tables and charts for more straightforward interpretation.

## Results

Out of 100 cases, the maximum number of cases were from the age group 30 to 39 years, i.e., 32 (32%), followed by the age group 20 to 29 years, i.e., 20 (20%), and the minimum from the age group more than 70 years, i.e., 01 (01%) case. Male cases outnumbered female cases, i.e., 85 (85%) vs. 15 (15%). The maximum number of subjects died in polytrauma, i.e., 47 (47%). Poisoning accounted for 26 cases (26%), followed by death due to disease with 16 cases (16%), while other causes, such as thermal injuries, hanging, and drowning, accounted for the remainder. Polytrauma is further subdivided into roadside accidents, falls from height, railway accidents, accidental cylinder bursts, and physical assault.

Among these, the majority are roadside accidents, followed by falls from height, railway accidents, and others. Further cases were categorised by hospital stay. The maximum number of patients were admitted to the hospital for less than 24 hours (43 cases out of 100), followed by brought-dead cases (30 cases); seven cases were between one and three days, 13 cases were between three and seven days, and seven cases were admitted for more than seven days. It is considered that the brought-dead cases did not receive any antimicrobial therapy, as shown in Table 1.

**Table 1:** Demographic Data of the Study Population

Parameter	Category	Number of Cases	Percentage (%)
Age Group (years)	20–29	20	20
	30–39	32	32
	≥70	1	1
	Other age groups	47	47
Sex	Male	85	85
	Female	15	15
Cause of Death	Polytrauma	47	47
	Poisoning	26	26
	Disease	16	16
	Others	11	11
Hospital Stay Duration	Brought dead	30	30
	< 24 hours	43	43
	1–3 days	7	7
	3–7 days	13	13
	> 7 days	7	7

A total of 61 single-organism culture-positive post-mortem heart blood samples were included for species-wise analysis. As shown in the study, *Klebsiella pneumoniae* was the most frequently isolated organism, accounting for 28 isolates (45.9%), followed by *Enterobacter *species in nine cases (14.8%) and *Acinetobacter baumannii* in eight cases (13.1%). *Escherichia coli* was isolated in six instances (9.8%), whereas *Staphylococcus aureus* was identified in four instances (6.6%). Less frequently isolated organisms included *Citrobacter* species and *Micrococcus* species, each accounting for two isolates (3.3%). *Enterococcus* species and Group D *Streptococcus* species were the least common, with one isolate each (1.6%) (Table 2).

**Table 2:** Distribution of Bacterial Isolates From Monomicrobial Post-mortem Heart Blood Cultures Overall distribution of post-mortem heart blood culture results. Of the 100 samples analysed, 66 were culture-positive, and 34 were sterile; among the positive samples, the species-wise analysis was restricted to monomicrobial cultures (n = 61), while polymicrobial cultures (n = 5) were excluded to avoid duplication and misrepresentation of percentages.

Bacterial Species	No. of Isolates	Percentage
Klebsiella pneumoniae	28	45.9
*Enterobacter* spp.	9	14.8
Acinetobacter baumannii	8	13.1
Escherichia coli	6	9.8
Staphylococcus aureus	4	6.6
*Citrobacter* spp.	2	3.3
*Micrococcus* spp.	2	3.3
*Enterococcus* spp.	1	1.6
Group D *Streptococcus* spp.	1	1.6
Total	61	100.0

Of the 66 culture-positive cases, 61 (92.4%) showed monomicrobial growth, while polymicrobial growth was observed in five cases (7.6%). Polymicrobial cultures were excluded from species-wise analysis to avoid duplication of isolates and misrepresentation of percentages and were interpreted cautiously due to the possibility of contamination or post-mortem translocation.

The association between cause of death and blood culture results is presented in Table 3. Among cases of polytrauma, blood cultures were positive in 32 cases (68.1%) and sterile in 15 cases (31.9%). In poisoning-related deaths, equal proportions of positive and sterile cultures were observed (13 cases each; 50.0%). Thermal injuries showed positive cultures in four cases (44.4%) and sterile cultures in five cases (55.6%). All cases of hanging and drowning demonstrated positive blood cultures (100% each), although the sample size in these categories was limited. In disease-related deaths, 15 cases (93.8%) yielded positive cultures, while only one case (6.2%) was sterile. Overall, a statistically significant association was observed between cause of death and blood culture results (chi-square = 11.442, df = 5, P = 0.043).

**Table 3:** Association Between the Cause of Death Categories and Blood Culture Result ^a ^Pearson’s chi-square test. Values are expressed as numbers (percentages). The chi-square test was used to assess the association between the cause of death and blood culture results. Chi-square = 11.442, df = 5, P = 0.043 (statistically significant). Gram-negative organisms predominated in polytrauma and disease-related deaths, whereas Gram-positive isolates were infrequent across all categories.

Cause of Death Categories	Culture Positive n (%)	Culture Sterile n (%)	Total n (%)	Chi-square (χ²)	df	P-value
Polytrauma	32 (68.1)	15 (31.9)	47 (47.0)	11.442ᵃ	5	0.043
Poisoning	13 (50.0)	13 (50.0)	26 (26.0)			
Thermal injuries	4 (44.4)	5 (55.6)	9 (9.0)			
Hanging	1 (100.0)	0 (0.0)	1 (1.0)			
Drowning	1 (100.0)	0 (0.0)	1 (1.0)			
Disease	15 (93.8)	1 (6.2)	16 (16.0)			
Total	66 (66.0)	34 (34.0)	100 (100)			

Table 4 demonstrates the relationship between the time interval from death to autopsy and blood culture results. Among cases autopsied within six hours of death, five cases (83.3%) showed positive cultures. For the 6-12-hour interval, 12 cases (63.2%) were culture-positive, while seven cases (36.8%) were sterile. In the 12-24-hour interval group, positive cultures were obtained in 49 cases (65.3%), with 26 cases (34.7%) remaining sterile. No statistically significant association was found between the time interval from death to autopsy and blood culture positivity (chi-square = 0.887, df = 2, P = 0.642).

**Table 4:** Association Between Post-mortem Interval and Blood Culture Results ^a^ Pearson’s chi-square test.

Post-mortem Interval (Hours)	Culture Positive n (%)	Culture Sterile n (%)	Total n (%)	Chi-square (χ²)	df	P-value
< 6 hours	5 (83.3)	1 (16.7)	6 (6.0)	0.887ᵃ	2	0.642
6–12 hours	12 (63.2)	7 (36.8)	19 (19.0)			
12–24 hours	49 (65.3)	26 (34.7)	75 (75.0)			
Total	66 (66.0)	34 (34.0)	100 (100)			

The association between hospital stay duration and blood culture results was assessed using the chi-square test and was not statistically significant (χ² = 0.320, df = 4, P = 0.988), as shown in Table 5. Of the 100 post-mortem heart blood samples, 66 showed bacterial growth, and 34 were sterile; species-wise analysis was performed only on the 61 monomicrobial culture-positive cases.

**Table 5:** Association Between Duration of Hospital Stay and Blood Culture Results ^a^ Pearson’s chi-square test.

Hospital Stay Duration	Culture Positive n (%)	Culture Sterile n (%)	Total n (%)	Chi-square (χ²)	df	P-value
Less than 24 hours	28 (65.1)	15 (34.9)	43 (43.0)	0.320ᵃ	4	0.988
1–3 days	5 (71.4)	2 (28.6)	7 (7.0)			
3–7 days	8 (61.5)	5 (38.5)	13 (13.0)			
> 7 days	5 (71.4)	2 (28.6)	7 (7.0)			
Brought dead	20 (66.7)	10 (33.3)	30 (30.0)			
Total	66 (66.0)	34 (34.0)	100 (100)			

## Discussion

To maintain uniformity and remove age bias, the present study included medico-legal cases from various age groups among the 100 autopsy cases, of which 32% of cases belonged to the 30-39 years age group, 20% to the 20-29 years age group, and 18% to the 40-49 years age group. The average age of the cases ranged from 38.76 to 13.99 years.

In the study published by Koneman et al., ages ranging from the second to the ninth decades of life were included, with the highest frequencies in the fifth, sixth, and seventh decades [5]. In the present study, participants' ages were younger than those in other studies. This difference in age range can be explained on the basis that most cases of polytrauma were included in the research, and the younger population is more prone to getting involved in accidents. Lalwani et al. reported similar age-range trends in their study of ventilator-associated pneumonia cases. The age of the patients ranged from nine months to 82 years (average 37.4 years). The study found no statistically significant association between hospital stay length and culture results [11]. A study by Koneman et al. in 1971 looked at 91 autopsies of patients, 46 of whom showed signs of infection and had received antibiotics before they died [5]. We also evaluated the effect of pre-mortem antibiotic therapy on the ability to isolate bacteria post-mortem. There was no significant difference in the percentages of patients with positive cultures between the treated (90%) and non-treated (84%) groups.

In the present study, the average hospital stay was one to three days, with 50% of cases lasting up to three days. A total of 30 cases were brought dead to the hospital. The cases that were brought dead were considered untreated, i.e., no antimicrobial therapy was administered in those cases. Patients admitted to the hospital for less than three days showed a similar percentage of positive blood culture results as those who had stayed for more than seven days. Thus, we conclude that the duration of hospital stay was not associated with the blood culture result, indicating that the timing of hospitalisation had no bearing on post-mortem cultures in our study.

In our study, out of 100 cultures from heart blood samples, 66 exhibited organism growth, while the remaining 34 showed no increase. Koneman et al. reported similar findings, obtaining 1285 cultures from various organs in 396 consecutive autopsies. In these 1285 cultures, 28% were sterile. However, if only heart blood is considered, only 53% of blood cultures yielded organisms, and the remaining 47% were sterile [5,6].

In the present study, 61 cultures (92.4%) yielded growth of only one organism, and six (7.6%) yielded growth of more than one organism. However, Koneman et al. (1971) reported that among culture samples that grew, 74% yielded a single organism, and the remaining 26% yielded two or more organisms [5]. The high proportions of cases in which either no growth (28.5%) or a single species (42.0%) was recovered substantiate the validity of the technique and the care taken in obtaining cultures.

In the present study, the most common species isolated among positive cultures was *Klebsiella pneumoniae*; 28 out of 66 positive cultures (42.4%) yielded *Klebsiella pneumoniae*, followed by *Enterobacter* species (9/66 cases) and *Acinetobacter baumannii* (8/66 cases), accounting for 13.6% and 13.1%, respectively. The remaining cultures yielded organisms such as *E. coli *(6/66 cases), *Staphylococcus aureus* (4/66 cases), and *Citrobacter* species (2/66 cases). Similar results, with *Klebsiella pneumoniae* among the commonly isolated organisms in post-mortem blood cultures, were reported by Demir et al. [19]. In their study, out of 21 positive samples, five samples yielded *Klebsiella pneumoniae*, followed by *Pseudomonas aeruginosa* (3/21 samples) and *E. coli* (2/21 samples). Less frequently isolated organisms, such as Micrococcus spp. and Group D Streptococcus, were considered potential contaminants or incidental findings and were interpreted in conjunction with autopsy findings and clinical context.

In a 2016 study, Sunagawa et al. [3] reported that among 23 bacterial isolates detected in 18 autopsy cases, *Escherichia coli* was the most frequently identified organism (five cases, 27.8%), followed by *Staphylococcus aureus* and *Klebsiella pneumoniae*. In the present study, the cause of death was a significant factor in determining whether the culture would yield any microorganisms. Blood cultures showed bacterial growth in almost all samples obtained from subjects who died of the disease. This was followed by cases of polytrauma, in which bacterial isolates were detected in more than two-thirds of cases. The association between the cause of death and culture result was found to be statistically significant. It can be implied from the results obtained that the chances of getting a positive culture are higher in polytrauma cases and death due to disease in comparison to hanging or drowning.

In the present study, the time gap between death and autopsy is within 24 hours. In the majority of the patients (75%), an autopsy was done between 12 and 24 hours post-death. Kurtin reported similar findings in his study, noting that bacterial contamination is not a significant factor in the first 48 hours after death [12]. Similar findings were reported by Riedal in 2014, who stated that the theory of agonal spread has very little evidence; post-mortem bacterial transmigration may have little influence on post-mortem microbiological cultures if the autopsy is performed within 24 to 48 hours after death [7-11]. In the present study, no statistically significant association was observed between post-mortem blood culture results and either the time interval between death and autopsy or the duration of hospital stay; however, these findings should be interpreted cautiously, particularly with respect to possible effects of pre-mortem antibiotic use and the limited sample size.

Numerous studies have highlighted the importance of blood cultures and post-mortem microbiology in supporting the diagnosis of infection and sepsis, provided they are interpreted cautiously in forensic settings. There have been studies done that emphasised the challenges posed by post-mortem changes, bacterial translocation, and the lack of definitive biomarkers, underscoring the need for integrated interpretation of microbiological findings. Other authors have similarly noted that post-mortem bacteriology provides valuable adjunctive information, particularly in cases of sudden or unexplained death, when correlated with autopsy findings and clinical context [13-18]. Pre-mortem antibiotic exposure, particularly among hospitalised cases, may have influenced culture positivity or negativity; however, detailed documentation regarding the type and duration of antimicrobial therapy was not uniformly available.

Thus, in this study, which included a balanced number of participants aged 10 to 70, mostly men, the only factor strongly linked to the heart blood culture results was how the person died. Other factors, like how long they stayed in the hospital and the time between death and the autopsy, did not show a significant connection to the heart blood culture results. The results of the heart blood culture did not significantly correlate with other factors, such as the duration of the hospital stay and the time interval between death and autopsy.

Limitations

This single-centre, cross-sectional study with a relatively small sample size limits generalisability and may reflect institutional referral patterns. Information on pre-mortem antibiotic use, especially in brought-dead cases, was incomplete and could have influenced culture results, while reliance on heart blood samples alone, low bacterial load, possible contamination, agonal spread, post-mortem translocation, and lack of molecular confirmation further limit interpretation. The study could not assess comorbidities, immune status, histopathological evidence of sepsis, perform multivariate analysis, or reliably determine time to culture positivity, and small subgroup sizes precluded meaningful confidence intervals; nevertheless, given the limited global data on post-mortem blood cultures in unnatural deaths, the study helps address an important forensic knowledge gap.

## Conclusions

This cross-sectional observational study analysed 100 cases of unnatural death, with polytrauma as the most common cause, followed by poisoning and disease-related deaths. Of the post-mortem heart blood samples, 66 showed predominantly monomicrobial bacterial growth, with *Klebsiella pneumoniae* as the most frequent isolate, followed by *Enterobacter* spp. and *Acinetobacter baumannii*. Culture positivity showed a significant association with cause of death, whereas hospital stay and post-mortem interval did not. When interpreted alongside autopsy findings, post-mortem blood cultures may provide supportive evidence in suspected infection-related deaths; the predominance of Gram-negative organisms, particularly in trauma and disease-related deaths, may reflect stress-related translocation or systemic compromise rather than true primary infection. Given the limited global data on post-mortem blood culture profiles in unnatural deaths, more research using large-scale, multicentre studies with molecular techniques and multiple sterile sampling sites is needed.
